# Mechanisms and Therapeutic Perspectives of Podocyte Aging in Podocytopathies

**DOI:** 10.3390/ijms26189159

**Published:** 2025-09-19

**Authors:** Si-Jia Ma, Yu-Ting Zhu, Fang-Fang He, Chun Zhang

**Affiliations:** Department of Nephrology, Union Hospital, Tongji Medical College, Huazhong University of Science and Technology, Wuhan 430022, China; masijia1018@163.com (S.-J.M.); drzhuyuting@hust.edu.cn (Y.-T.Z.)

**Keywords:** podocyte aging, podocytopathies, proteinuria, glomerulosclerosis

## Abstract

Podocytes are highly specialized, terminally differentiated epithelial cells essential for maintaining the glomerular filtration barrier. Their limited regenerative capacity and high metabolic demands render them particularly susceptible to aging-related stress. Accumulating evidence indicates that podocyte aging, characterized by cellular senescence, mitochondrial dysfunction, autophagy impairment, and epigenetic alterations, significantly contributes to the pathogenesis of diverse glomerular diseases collectively termed podocytopathies. These include focal segmental glomerulosclerosis, membranous nephropathy, minimal change disease, diabetic kidney disease, and lupus nephritis. This review discusses the cellular and molecular mechanisms driving podocyte aging and explores how these alterations predispose to podocyte injury, loss, and dysfunction, ultimately culminating in podocytopathies. Furthermore, we highlight current and emerging therapeutic strategies that aim to preserve podocyte health by targeting aging-associated pathways. Understanding podocyte aging elucidates mechanisms of chronic kidney disease progression and identifies novel therapeutic strategies for age-specific interventions in podocytopathies.

## 1. Introduction

Podocytes are highly specialized and terminally differentiated epithelial cells that line the outer aspect of the glomerular basement membrane (GBM). They constitute a critical component of the glomerular filtration barrier (GFB) [1]. Podocyte injury, manifested as foot process effacement (FPE), detachment, or apoptosis, directly compromises GFB integrity, leading to proteinuria. Crucially, the limited proliferative capacity positions podocytes as central determinants in the pathogenesis of proteinuria [2].

Podocyte aging is characterized by irreversible cell cycle arrest, mitochondrial dysfunction, and acquisition of a senescence-associated secretory phenotype (SASP) [3]. SASP entails hypersecretion of pro-inflammatory cytokines, chemokines, and proteases, propagating local inflammation and tissue damage. This process is accelerated in podocytopathies, contributing to FPE and podocyte detachment [4]. Podocytopathies such as focal segmental glomerulosclerosis (FSGS), membranous nephropathy (MN), minimal change disease (MCD), diabetic kidney disease (DKD), and lupus nephritis (LN) converge on the critical endpoint of podocyte loss [5]. Cellular senescence significantly exacerbates podocyte vulnerability to stimuli, highlighting the central role of podocyte aging in the pathophysiology of podocytopathies.

Understanding podocyte aging is, therefore, crucial for elucidating the mechanisms of podocytopathy progression. Senescent podocyte accumulation in aging kidneys correlates strongly with disease severity and proteinuria degree [6]. SASP from podocyte aggravates glomerular inflammation, endothelial dysfunction, and interstitial fibrosis [7]. Importantly, podocyte aging often precedes overt histopathological changes, rendering it both a potential early biomarker and a promising therapeutic target for halting podocytopathy progression [3]. Given the escalating global health burden of podocytopathies, elucidating mechanisms of podocyte aging is very important.

In this review, we discuss recent insights into podocyte aging in podocytopathies and explore novel therapeutic strategies.

## 2. Podocyte Structure and Function

Podocytes are terminally differentiated epithelial cells that form the visceral layer of Bowman’s capsule and are indispensable for the structural and functional integrity of GFB. Their primary processes branch into interdigitating secondary foot processes that envelop glomerular capillaries. Adjacent foot processes are bridged by the slit diaphragm (SD), which is an evolutionarily conserved, modified adherens junction forming a zipper-like extracellular ultrafiltration sieve with precise size- and charge-selectivity [8]. The SD is dynamically maintained by a cytoskeleton composed of actin stress fibers anchored to the GBM via focal adhesion complexes [9]. Key functional proteins of SD, including nephrin, podocin, and synaptopodin, ensure perm-selective barrier function, preventing proteinuria [10,11,12]. Critically, podocytes exhibit limited replicative capacity due to the high expression of cyclin-dependent kinase inhibitors, which enforce cell cycle arrest [13]. This post-mitotic state, combined with high metabolic activity and exposure to mechanical stress and circulating toxins, renders podocytes highly vulnerable to injury or aging. Consequently, podocyte loss is largely irreversible, triggering proteinuria and progressive glomerulosclerosis [2].

## 3. Cellular and Molecular Mechanisms of Podocyte Aging

### 3.1. Cellular Senescence

**Figure 1:**
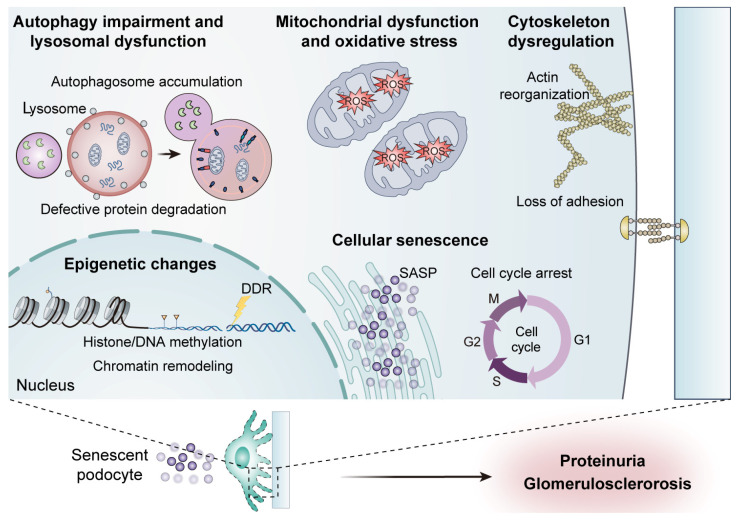
Key pathophysiological mechanisms driving podocyte aging. Podocyte aging is a complex process characterized by several interconnected pathways. Cellular senescence manifests as irreversible cell cycle arrest, persistent DDR, and secretion of a SASP, promoting local inflammation and paracrine senescence. Mitochondrial dysfunction and oxidative stress, including impaired oxidative phosphorylation, accumulation of damaged mitochondria, excess ROS production, and mtDNA damage, compromise energy homeostasis and activate redox-sensitive stress signaling. Autophagy and lysosomal impairment lead to defective autophagosome formation, lysosomal dysfunction, and accumulation of misfolded proteins and damaged organelles, exacerbating proteotoxic stress. Epigenetic alterations, such as DNA and histone methylation, and chromatin remodeling, reshape gene expression programs that control stress responses, mitochondrial function, and cell cycle. Cytoskeletal dysregulation, particularly affecting actin filament organization, SD integrity, and adhesion to GBM, results in FPE and increased podocyte detachment. Together, these pathways converge to compromise podocyte function, reduce regenerative capacity, and drive progressive glomerular injury across diverse podocytopathies. Abbreviation: DDR: DNA damage response, SASP: senescence-associated secretory phenotype, ROS: reactive oxygen species, mtDNA: mitochondrial DNA, SD: slit diaphragm, GBM: glomerular basement membrane, FPE: foot process effacement.

Cellular senescence, a key hallmark of aging, critically contributes to the functional deterioration of podocytes [14]. Senescent podocytes exhibit permanent cell cycle arrest, resistance to apoptosis, and distinct morphological and biochemical alterations, including hypertrophy and cytoskeletal simplification [6,14] (Figure 1). A primary driver of podocyte aging is telomere attrition [15]. Repeated cell division and oxidative stress cause telomeres to shorten over time. This eventually triggers a persistent DNA damage response (DDR) [16]. In podocytes, telomere dysfunction induces genomic instability and potently activates the p53-p21^Cip1^ and p16^INK4a^-Rb pathways, enforcing irreversible cell cycle arrest [3,6].

Additionally, senescent podocytes acquire an SASP, characterized by hypersecretion of pro-inflammatory cytokines, chemokines, growth factors, and matrix-remodeling enzymes [17]. The SASP fosters a pro-fibrotic and pro-inflammatory microenvironment within the glomerulus, exacerbating tissue injury and accelerating disease progression [7,18]. In aging kidneys, glomeruli exhibit elevated expression of senescence markers, including p16^INK4a^ and senescence-associated β-galactosidase (SA-β-gal), which correlates with reduced podocyte density and albuminuria [6]. Notably, the DDR not only sustains the senescent state but also perpetuates SASP signaling through persistent activation of ATM/ATR kinases and downstream NF-κB transcription factors [17].

Collectively, telomere shortening, DDR activation, and SASP propagate a self-amplifying circuit that exacerbates podocyte dysfunction and significantly contributes to the pathogenesis of age-related glomerulopathies.

### 3.2. Mitochondrial Dysfunction and Oxidative Stress

Mitochondrial dysfunction constitutes a hallmark feature of senescent podocytes (Figure 1). Pronounced mitochondrial structural damage, including cristae dissolution and outer membrane rupture, was revealed in the glomeruli of aged mice. This was accompanied by upregulated expression of reactive oxygen species (ROS)-producing enzymes and diminished levels of oxidative phosphorylation complexes I–IV [19,20]. Metabolic profiling of senescent podocytes demonstrates a shift from mitochondrial respiration toward glycolytic dependence and impaired fatty acid oxidation [19]. Mechanistically, defects in the electron transport chain coupled with NADPH oxidase hyperactivity propel excessive ROS production [21]. Elevated mitochondrial ROS (mtROS) inflict damage to mitochondrial DNA (mtDNA), lipids, and proteins, precipitating mitochondrial permeability transition pore (mPTP) opening, cytochrome c release, caspase activation, and podocyte apoptosis [22].

Key regulators of mitochondrial integrity, including sirtuin 1 (SIRT1), PPARγ coactivator-1α (PGC-1α), and mitochondrial transcription factor A (TFAM), exhibit downregulation in senescent podocytes [23,24]. In aldosterone-infusion models and human nephropathies, reduced ATP production, collapsed mitochondrial membrane potential, decreased mtDNA copy number, and ultrastructural abnormalities precede podocyte loss [21,25]. Pharmacological activation of peroxisome proliferator-activated receptor Gamma (PPARγ) or SIRT1 restores mitochondrial function and attenuates ROS generation, preserving podocyte viability [25,26].

In summary, podocyte mitochondrial dysfunction manifests as compromised bioenergetics, oxidative stress, impaired quality control, and activation of cell death pathways, establishing a self-amplifying cycle that accelerates glomerular aging.

### 3.3. Autophagy Impairment and Lysosomal Dysfunction

Autophagy serves as an essential quality-control mechanism in terminally differentiated podocytes, facilitating the clearance of damaged proteins and organelles to maintain cellular homeostasis [27,28] (Figure 1). Genetic ablation of the core autophagy gene autophagy-related 5 (Atg5) in podocytes induces proteinuria and accelerates glomerulosclerosis. It also leads to hallmark aging phenotypes, including lipofuscin accumulation and ubiquitinated protein aggregates [29,30]. This confirms that impaired autophagy compromises cytoplasmic renewal and directly precipitates progressive podocyte aging.

Furthermore, lysosomal dysfunction results in autophagosome accumulation, endoplasmic reticulum stress (ERS), FPE, and premature lethality in murine models [31,32]. Similarly, podocyte-specific knockout of the prorenin receptor or lysosomal proteases contributes to albuminuria, cytoskeletal disruption, and eventual glomerulosclerosis, underscoring the critical importance of lysosomal integrity to podocyte [33,34].

The autophagy-lysosome pathway exhibits functional crosstalk with the ubiquitin-proteasome system (UPS) [35]. Autophagy deficiency initially upregulates proteasomal activity, but ultimately leads to the accumulation of cytotoxic protein aggregates, exacerbating podocyte injury [32]. Intriguingly, selective induction or enhancement of autophagy via rapamycin or AMP-activated protein kinase (AMPK)/SIRT1 activators exerts protective effects on podocytes. However, excessive autophagic activity may exert detrimental effects in specific contexts [36,37].

In summary, compromised autophagic flux and lysosomal dysfunction in podocytes establish a vicious cycle characterized by misfolded protein accumulation, chronic stress response activation, cytoskeletal destabilization, and cell death.

### 3.4. Epigenetic Changes

Podocytes undergo profound epigenetic remodeling during aging and in disease states, significantly impacting gene expression, chromatin architecture, and cellular identity [38] (Figure 1). These alterations encompass DNA methylation dynamics, histone post-translational modifications, and chromatin remodeling events, collectively disrupting podocyte homeostasis [39].

Histone modifications critically influence podocyte fate. Aged podocytes exhibit reduced trimethylation of histone H3 lysine 27 (H3K27me3), attributable to decreased expression of the methyltransferase enhancer of zeste homolog 2 (EZH2). This imbalance drives transcriptional derepression of pro-senescence and pro-fibrotic genes, contributing to oxidative stress, FPE, and podocyte injury [40,41]. Dysregulation of histone deacetylases (HDACs), particularly HDAC1/2 and SIRT1/6, further exacerbates genomic instability. Podocyte-specific ablation of Hdac1/2 or Sirt1 induces proteinuria and accelerates glomerulosclerosis [42,43,44].

DNA methylation alterations prominently feature in podocyte pathology [45]. Under hyperglycemic or oxidative stress, DNA methyltransferases (DNMTs) are recruited to promoters of essential podocyte genes, inducing transcriptional silencing that disrupts SD integrity and promotes proteinuria [39]. DNMT1-mediated repression may establish an epigenetic memory, perpetuating injury responses and impairing regenerative capacity [46]. Chromatin architecture is progressively destabilized with aging. Senescent podocytes exhibit loss of constitutive heterochromatin and form senescence-associated heterochromatin foci, driven by altered histone landscapes and diminished HDAC activity. This reorganization compromises genomic integrity and reinforces SASP [42].

In summary, aging- and disease-induced epigenetic dysregulation, manifested through histone/DNA methylation defects and chromatin disorganization, disrupts core podocyte transcriptional networks, promoting senescence, dedifferentiation, and attrition of podocytes.

### 3.5. Cytoskeleton Dysregulation

Podocyte foot processes rely on a precisely regulated actin cytoskeleton, anchored by SD complexes and integrin-based adhesions, to withstand hemodynamic stress and maintain GFB integrity [47]. Under the condition of aging and diseases, this actin network undergoes destabilization, precipitating FPE and weakened adhesion to the GBM, and eventually podocyte detachment [48,49] (Figure 1).

A core mechanism involves diminished expression of SD proteins, notably nephrin (NPHS1) and podocin (NPHS2) [50]. Transcriptional downregulation of these canonical genes in aged podocytes attenuates critical linkages between the SD, focal adhesions, and the actin cytoskeleton [51]. FPE, a hallmark of aging and glomerulopathies, results from dynamic actin reorganization and synaptopodin disassembly, reflecting compromised cytoskeletal stability [52].

Genetic and acquired defects in actin-regulating proteins, such as α-actinin-4 (ACTN4), inverted formin-2 (INF2), and non-muscle myosin heavy chain 9 (MYH9), contribute to aberrant actin architecture [50]. For instance, ACTN4 and INF2 mutations impair actin filament crosslinking and resilience, diminishing podocyte capacity to recover from mechanical stretch and promoting detachment under physiological stress [53,54].

Podocyte–GBM adhesion, mediated primarily by α3β1 integrins and focal adhesion components like integrin-linked kinase (ILK), is disrupted in aging. Consequently, this cascade impairs focal adhesion turnover, stress fiber formation, and lamellipodial dynamics, culminating in podocyte detachment and proteinuria [49]. Furthermore, integrin downregulation and loss of adhesion receptors progressively weaken podocyte anchorage to the GBM [55].

Collectively, aging drives progressive structural deterioration of SD complexes, actin filament organization, and adhesion machinery, ultimately manifesting as FPE and podocyte loss.

## 4. Podocyte Aging in Podocytopathies

### 4.1. Focal Segmental Glomerulosclerosis

FSGS is a heterogeneous podocytopathy characterized by podocyte loss, FPE, and segmental glomerular scarring, often progressing to proteinuria and end-stage kidney disease [56]. Genetic causes, complement-mediated injury, hemodynamic and metabolic stress, and aberrant signaling pathways all converge to injure podocytes, rendering them vulnerable to senescence and detachment [56,57].

Mitochondrial dysfunction and metabolic derangement are central to podocyte aging in FSGS [21]. High-risk apolipoprotein L1 (APOL1) variants impair mitochondrial fatty acid oxidation, causing lipid intermediate accumulation, ATP depletion, and increased susceptibility to cytoskeletal instability and detachment. In addition, APOL1-mediated mitochondrial membrane disruption induces mPTP opening and mtROS overproduction, leading to oxidative damage of mtDNA and respiratory chain components [58]. This oxidative stress activates redox-sensitive pathways and enforces permanent cell cycle arrest. Meanwhile, sustained hyperactivation of rapamycin complex 1 (mTORC1), reflected by increased S6K1 and 4E-BP1 phosphorylation, promotes podocyte hypertrophy and anabolic overload by aggravating mitochondrial stress, ERS, and proteotoxicity [59].

Autophagy impairment and aberrant cell cycle reentry are key drivers of podocyte aging in FSGS [27]. Hyperactivation of the rapamycin (mTOR)/S6K1 axis by methyltransferase-like 14 (METTL14)-mediated m^6^A methylation suppresses SIRT1, a central regulator of autophagy and mitochondrial quality control [60]. Consequently, defective autophagosome formation hampers the clearance of damaged mitochondria, oxidized proteins, and cytoskeletal aggregates, leading to chronic ERS and enhanced DDR, which reinforce senescence signaling. In parallel, aberrant expression of mitotic arrest deficient 2-like protein 2 (MAD2B) induces forced cell cycle reentry, culminating in mitotic catastrophe and ultimately podocyte loss [61].

Inflammatory and complement-mediated mechanisms critically reinforce podocyte aging in FSGS. Activation of the complement component C5a/C5a receptor 1 (C5aR1) axis promotes inflammation, apoptosis, and a senescence-like phenotype, driving podocyte detachment and GFB disruption, while C5aR1 inhibition alleviates injury and disease progression [62]. Similarly, podocyte protease receptor 1 (PAR-1) stimulation induces stress, cell cycle dysregulation, and senescence signaling, leading to podocyte dysfunction and glomerulosclerosis [63]. In addition, inflammatory cues upregulate C–C chemokine receptor type 2 (CCR2) in podocytes, enhancing monocyte/macrophage recruitment, chronic inflammation, and local immune infiltration [64]. This inflammatory burden exacerbates podocyte injury, redox imbalance, and DDR, thereby consolidating the senescent phenotype.

Cytoskeletal disruption and mechano-transductive stress exacerbate podocyte fragility. Loss of key structural proteins, including α3β1 integrin, Crumbs homolog 2 (Crb2), and FAM40A, together with dysfunction of actin regulators such as Rho-associated coiled-coil containing protein kinase 2 (ROCK2) and focal adhesion kinase (FAK), disrupts cytoskeletal organization and SD stability [52,65,66]. In parallel, nuclear exclusion of yes-associated protein (YAP), often induced by biomechanical stress, suppresses gene programs required for cytoskeletal repair and mechanosensing, leading to FPE, SD breakdown, and weakened GBM adhesion [67]. This structural vulnerability exposes podocytes to aberrant mechanical forces, which activate senescence-related pathways, impair repair capacity, and accelerate podocyte detachment and senescence [52].

Epigenetic and post-translational dysregulation consolidate podocyte senescence in FSGS. METTL14-mediated m^6^A modification of SIRT1 transcripts represses this longevity-associated deacetylase, impairing oxidative stress resistance, DNA repair, and mitochondrial function, and disrupting chromatin maintenance [60]. Under chronic glomerular stress, transient receptor potential canonical 6 (TRPC6) activation with downstream S6 kinase phosphorylation drives compensatory hypertrophy, which imposes excessive metabolic and biosynthetic load, exacerbating replication stress and senescence [59]. Collectively, these events reshape podocyte transcriptional and chromatin landscapes, locking cells into a senescent state that perpetuates glomerular injury.

**Figure 2:**
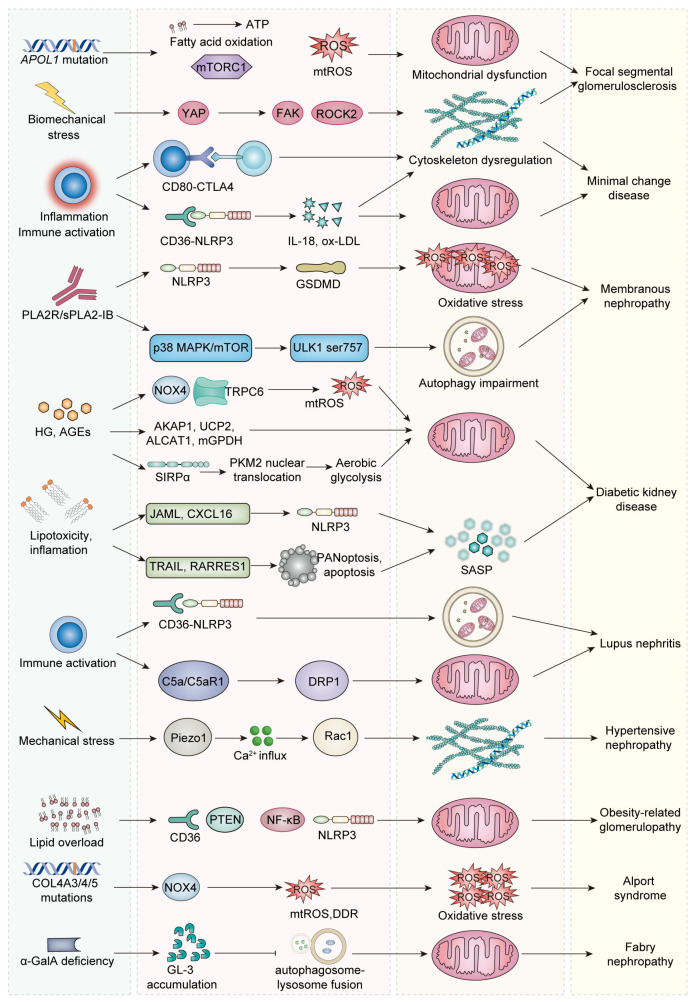
Cross-disease map linking stimuli, signaling pathways, aging phenotypes, and podocytopathies. The first column summarizes upstream stimuli; the second shows representative signaling nodes; the third denotes convergent cellular aging phenotypes, and the last column indicates diseases. In FSGS and MCD, APOL1 risk signaling, biomechanical stress, and immune-inflammatory cues converge on cytoskeletal dysregulation and mitochondrial dysfunction. In MN, PLA2R/sPLA2-IB activates p38MAPK/mTOR, leading to ULK1 Ser757 phosphorylation and inhibition of autophagy. In LN, immune activation via CD36 and C5a/C5aR1 promotes DRP1 phosphorylation, combining autophagy impairment with mitochondrial injury. Mechanical stress sensed by Piezo1 perturbs the actin cytoskeleton in HN. Lipid overload activates NF-κB/NLRP3, driving mitochondrial dysfunction in ORG. In DKD, HG/AGEs trigger NOX4/TRPC6-dependent mtROS and mitochondrial injury; altered AKAP1, UCP2, ALCAT1, and mGPDH further compromise mitochondrial quality control, while SIRPα-induced PKM2 nuclear translocation favors aerobic glycolysis. Lipotoxic/inflammatory mediators activate NLRP3 and a SASP; TRAIL and RARRES1 promote PANoptosis/apoptosis, reinforcing podocyte senescence. In AS, COL4A3/4/5 mutations upregulate NOX4, increasing mtROS and DDR to produce oxidative stress; α-galactosidase A deficiency in Fabry nephropathy causes GL-3 accumulation and defective autophagosome–lysosome fusion, culminating in mitochondrial dysfunction. Abbreviations: APOL1: apolipoprotein L1, PLA2R: phospholipase A2 receptor, sPLA2-IB: secretory phospholipase A2 group IB, ATP: adenosine triphosphate, mTORC1: mechanistic target of rapamycin complex 1, ROS: reactive oxygen species, YAP: yes-associated protein, FAK: focal adhesion kinase, ROCK2: Rho-associated kinase 2, CTLA4: cytotoxic T-lymphocyte antigen 4, IL-18: interleukin 18, ox-LDL: oxidized low-density lipoprotein, NLRP3: NOD-like receptor family pyrin domain-containing 3, GSDMD: Gasdermin D, MAPK: mitogen-activated protein kinase, mTOR: mechanistic target of rapamycin, C5a: complement component C5a, C5aR1: C5a receptor 1, ULK1: Unc-51-like kinase 1, DRP1: dynamin-related protein 1, NOX4: NADPH oxidase 4, TRPC6: transient receptor potential canonical 6, mtROS: mitochondrial reactive oxygen species, DDR: DNA-damage response, AKAP1: A-kinase anchoring protein 1, UCP2: uncoupling protein-2, ALCAT1: acyl-CoA:lysocardiolipin acyltransferase-1, mGPDH: mitochondrial glycerol-3-phosphate dehydrogenase, SIRPα: signal regulatory protein-α, PKM2: pyruvate kinase M2, JAML: junctional adhesion molecule-like, CXCL16: C–X–C chemokine ligand 16, RARRES1: retinoic-acid-responsive 1, TRAIL: TNF-related apoptosis-inducing ligand, PANoptosis: integrated pyroptosis–apoptosis–necroptosis, SASP: senescence-associated secretory phenotype, GL-3: globotriaosylceramide, PTEN: phosphatase and tensin homolog, HG: hyperglycemia, AGEs: advanced glycation end-products, ORG: obesity-related glomerulopathy, HN: hypertensive nephropathy, MCD: minimal change disease, MN: membranous nephropathy, LN: lupus nephritis, FSGS: focal segmental glomerulosclerosis, DKD: diabetic kidney disease, AS: Alport syndrome.

**Figure 3:**
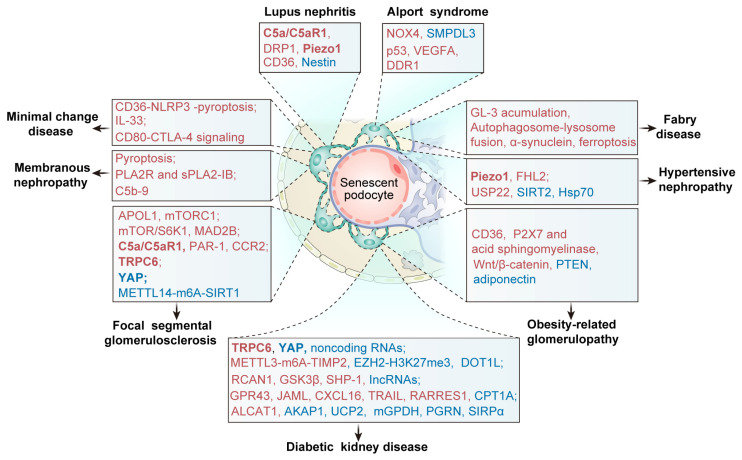
Key molecular players driving podocyte aging in podocytopathies. A variety of key molecules are implicated in the progression of podocyte aging across multiple podocytopathies, including focal segmental glomerulosclerosis, minimal change disease, membranous nephropathy, diabetic kidney disease, lupus nephritis, Alport syndrome, Fabry disease, hypertensive nephropathy, and obesity-related glomerulopathy. These molecules contribute to distinct pathological mechanisms such as cellular senescence, mitochondrial dysfunction, impaired autophagy, epigenetic alterations, cytoskeletal dysfunction, and inflammatory responses. The molecules in red are upregulated in the disease condition, while the blue ones are downregulated. Abbreviation: C5a: complement component C5a, C5aR1: C5a receptor 1, DRP1: dynamin-related protein 1, NOX4: NADPH oxidase 4, SMPDL3b: sphingomyelin phosphodiesterase acid-like 3b, VEGFA: vascular endothelial growth factor A, DDR1: discoidin domain receptor 1, NLRP3: NOD-like receptor thermal protein domain-associated protein 3, IL-33: interleukin 33, CTLA-4: cytotoxic T-lymphocyte antigen 4, PLA2R: phospholipase A2 receptor, sPLA2-IB: secretory phospholipase A2 group IB, C5b-9: complement component 5b-9, APOL1: apolipoprotein L1 gene, mTORC1: mechanistic target of rapamycin complex 1, mTOR: mechanistic target of rapamycin, MAD2B: mitotic arrest deficient 2-like protein 2, PAR-1: protease receptor 1, CCR2: C–C chemokine receptor type 2, TRPC6: transient receptor potential canonical 6, YAP: yes-associated protein, METTL14: methyltransferase-like 14, SIRT1: sirtuin 1, FHL2: four-and-a-half LIM domains protein 2, USP22: ubiquitin-specific protease 22, SIRT2: sirtuin 2, Hsp70: heat shock protein 70, P2X7: purinergic 2X7, PTEN: phosphatase and tensin homolog, METTL3: methyltransferase-like 3, TIMP2: tissue inhibitor of metalloproteinases 2, EZH2: enhancer of zeste homolog 2, H3K27me3: histone H3 lysine 27 trimethylation, DOT1L: disruptor of telomeric silencing 1-like, RCAN1: regulator of calcineurin 1, GSK3β: glycogen synthase kinase 3 beta, SHP-1: Src homology region 2 domain-containing phosphatase-1, GPR43: G protein-coupled receptor 43, JAML: junctional adhesion molecule-like protein, CXCL16: C–X–C chemokine ligand 16, TRAIL: tumor necrosis factor (TNF)-related apoptosis-inducing ligand, RARRES1: retinoic acid receptor responder protein-1, CPT1A: carnitine palmitoyltransferase-1A, ALCAT1: acyl-coenzyme A:lyso-cardiolipin acyltransferase-1, AKAP1: A-kinase anchoring protein 1, UCP2: uncoupling proteins 2, mGPDH: mitochondrial glycerol 3-phosphate dehydrogenase, PGRN: progranulin, SIRPα: signaling regulatory protein alpha.

In FSGS, podocyte aging arises from mitochondrial dysfunction, autophagy blockade, inflammation–complement activation, cytoskeletal disassembly, and epigenetic reprogramming (Figure 2 and Figure 3). Recognition of these intersecting mechanisms underscores potential interventions to delay podocyte aging and slow FSGS progression.

### 4.2. Minimal Change Disease and Membranous Nephropathy

MCD is an idiopathic podocytopathy and the leading cause of nephrotic syndrome, characterized by proteinuria, edema, and profound FPE [68]. Recent discoveries suggest an autoimmune component beyond classical podocyte injury [69,70,71,72].

Podocyte aging is central to the pathogenesis of MCD. Oxidative stress and lipid-driven inflammation activate the cluster of differentiation 36 (CD36)–NOD-like receptor thermal protein domain-associated protein 3 (NLRP3) inflammasome–pyroptosis cascade, elevating interleukin-18 (IL-18) and ox-LDL levels [73]. Nephrin autoantibody binding at the SD triggers nephrin phosphorylation and cytoskeletal remodeling, perpetuating podocyte stress [69]. Interleukin-33 (IL-33), via group 2 innate lymphoid cells (ILC2)-mediated type 2 T helper cells (Th2) cytokines, may exert protective effects but also contribute to inflammation and podocyte imbalance [71]. Dysregulated cluster of differentiation 80 (CD80)–cytotoxic T-lymphocyte antigen 4 (CTLA4) signaling further promotes cell cycle arrest, cytoskeletal disorganization, and mitochondrial dysfunction [70]. Collectively, these mechanisms enforce podocyte senescence, disrupt the GFB, and underlie the relapsing nature of MCD.

MN is an immune complex-mediated podocytopathy characterized by subepithelial immune deposits and complement activation, particularly C3 and C5b-9, which form the membrane attack complex (MAC) on podocytes and drive proteinuria [74,75]. Primary antigens such as phospholipase A2 receptor (PLA2R) drive this cascade, further exacerbated by C3a/C3aR-mediated podocyte injury [76,77].

Complement-mediated mitochondrial dysfunction initiates podocyte pyroptosis, dependent on ROS, caspase-1, Gasdermin D (GSDMD), and NLRP3 activation, which amplifies inflammatory signaling, SASP, and permanent cell cycle arrest [78]. In MN, impaired autophagy is driven by PLA2R and secretory phospholipase A2 group IB (sPLA2-IB) through p38MAPK/mTOR signaling, which phosphorylates unc-51 like autophagy activating kinase 1 (ULK1) at Ser757 and blocks AMPK–ULK1 interaction to inhibit autophagy initiation [79]. Insufficient autophagic flux causes accumulation of damaged mitochondria and misfolded proteins, aggravating oxidative stress and senescence. C5b-9 and lysosomal dysfunction further block autophagy, promoting cytoskeletal injury, SD disassembly, and podocyte detachment [80]. These events form a vicious cycle that accelerates podocyte aging and glomerular damage in MN.

Unlike FSGS, where metabolic stress dominates, podocyte senescence in MCD and MN is primarily shaped by immune dysregulation and complement activation, underscoring inflammatory-immune pathways as the main drivers (Figure 2 and Figure 3).

### 4.3. Diabetic Kidney Disease

Podocyte aging is a key pathological event in DKD. Podocytes from type 2 DKD patients show elevated senescence markers, correlating with proteinuria and hyperglycemia, indicating accelerated cellular senescence in the diabetic milieu [81,82].

Mitochondrial dysfunction and oxidative stress are fundamental drivers of podocyte aging in DKD. Hyperglycemia induces mtROS via the NADPH oxidase 4 (NOX4)/TRPC6 axis, causing oxidative stress, mtDNA damage, and ATP loss [83]. Dysregulation of mitochondrial regulators, including A-kinase anchoring protein 1 (AKAP1), uncoupling proteins 2 (UCP2), acyl-coenzyme A:lyso-cardiolipin acyltransferase-1 (ALCAT1), and mitochondrial glycerol 3-phosphate dehydrogenase (mGPDH), collectively disrupts mitochondrial integrity and energy metabolism. AKAP1 deficiency impairs mtDNA biogenesis [84]; UCP2 loss increases ROS and impairs autophagy [85]; pathological ALCAT1 upregulation generates peroxidized cardiolipin, promoting membrane instability and cytochrome c release [86]; and mGPDH loss disrupts the glycerophosphate shuttle and oxidative phosphorylation [87]. Protective progranulin (PGRN) normally facilitates mitophagy and biogenesis, but its loss causes damaged mitochondria to accumulate [88]. In parallel, reduced signaling regulatory protein α (SIRPα) enhances pyruvate kinase M2 (PKM2) nuclear translocation, shifting metabolism from oxidative phosphorylation to aerobic glycolysis, aggravating mitochondrial dysfunction and SASP activation [89]. Together, these mechanisms compromise podocyte viability, diminish regenerative capacity, and enforce senescence.

In DKD, autophagy impairment and lysosomal dysfunction drive podocyte aging. High glucose activates the phosphoinositide 3-kinase (PI3K)/protein kinase B (AKT)/mTORC1 pathway, inhibiting the ULK1/autophagy-related 13 (ATG13) complex and blocking autophagosome formation [90]. Concurrently, TRPC6-mediated calcium influx and calpain activation disrupt autophagic flux by impairing vesicle trafficking and lysosomal fusion [91]. YAP inhibition reduces Wilms tumor 1 (WT1) expression, suppressing autophagy-related genes [92], while noncoding RNAs such as circ-0000953 and CircHIPK3 interact with miR-665-3p to further repress autophagy networks [93,94]. These defects lead to the accumulation of dysfunctional mitochondria and p62-positive aggregates, compromising proteostasis and accelerating podocyte senescence [95].

Epigenetic and transcriptional dysregulation increase podocyte susceptibility to senescence in DKD. Accumulation of advanced glycation end-products (AGEs) suppresses EZH2, reducing H3K27me3 and activating pro-senescent transcriptional programs, while restoring EZH2 mitigates injury [96]. Methyltransferase-like 3 (METTL3)-mediated m^6^A modification stabilizes tissue inhibitor of metalloproteinases 2 (TIMP2) mRNA, enhancing stress signaling and podocyte injury, which is alleviated by METTL3 silencing [97]. Downregulation of disruptor of telomeric silencing 1-like (DOT1L) alters H3K79 methylation, therefore compromising genomic integrity [98]. Epigenetic upregulation of regulator of calcineurin 1 (RCAN1) activates calcineurin–nuclear factor of activated T cell (NFAT) signaling, promoting oxidative stress, inflammation, and cell cycle arrest [99]. LncRNAs, such as plasmacytoma variant translocation 1 (PVT1), HOXB3OS, and ENST00000436340, modulate pro-apoptotic transcripts, SIRT1 stability, and vesicular stress, respectively [100,101,102]. Additionally, glycogen synthase kinase 3 β (GSK3β) activation impairs β-catenin and nuclear factor erythroid 2-Related Factor 2 (NRF2) to weaken antioxidant defenses [103]. Increased Src homology region 2 domain-containing phosphatase-1 (SHP-1) activity reduces podocin SUMOylation, compromising SD integrity and cytoskeletal architecture [104]. Collectively, these alterations disrupt homeostasis and enforce podocyte senescence.

In DKD, podocyte aging is accelerated by lipotoxicity and chronic inflammation. Excess lipid accumulation, driven by dysregulated lipid transport and metabolism, disrupts mitochondrial β-oxidation and energy homeostasis and induces ERS. G protein-coupled receptor 43 (GPR43) activation enhances lipid uptake and triggers extracellular signal-regulated kinase (ERK)/early growth response 1 (EGR1) signaling, whereas its deficiency improves insulin sensitivity via AMPKα [105,106]. Loss of carnitine palmitoyltransferase-1A (CPT1A) further exacerbates lipotoxic stress and apoptosis [107]. Lipid overload activates the NLRP3 inflammasome, which is modulated by junctional adhesion molecule-like protein (JAML) and C–X–C chemokine ligand 16 (CXCL16), leading to IL-1β, IL-6, and TNF-α release and a pro-inflammatory microenvironment [108,109,110]. These cytokines activate downstream death pathways, including PANoptosis induced by tumor necrosis factor (TNF)-related apoptosis-inducing ligand (TRAIL) and death receptor 5, and apoptotic signaling amplified by retinoic acid receptor responder protein-1 (RARRES1) [111,112].

Compared with FSGS, DKD features additional systemic metabolic insults from hyperglycemia and lipid overload, which amplify oxidative stress and inflammatory signaling, highlighting the interplay between systemic metabolic derangements and podocyte senescence (Figure 2 and Figure 3).

### 4.4. Lupus Nephritis

LN features accelerated podocyte aging driven by immune complex deposition, complement activation, and chronic inflammation [113]. Sublytic assembly of C5b-9 on the membrane of podocyte induces mtROS generation and oxidative DNA damage, triggering irreversible cell cycle arrest [114,115].

In LN, impaired autophagy drives podocyte senescence and injury [116]. Nestin, an intermediate filament protein specific to podocytes, enhances mitophagy, clearing damaged mitochondria and reducing mtROS, thereby protecting podocytes from oxidative stress [117]. Conversely, CD36 upregulation suppresses autophagic flux, weakening cellular defenses [118]. While inflammatory stimuli such as interferon-α (IFN-α) and pathogenic antibodies can induce protective autophagy [119], inhibition or overwhelming of autophagy renders podocytes highly susceptible to irreversible senescence.

Inflammasome activation contributes to podocyte aging in LN [120]. CD36 inhibits autophagy and activates the NLRP3 inflammasome, promoting IL-1β release and SASP-driven local inflammation, which induces paracrine senescence in neighboring glomerular cells [118]. C5a/C5aR1 triggers mitochondrial stress and amplifies inflammation, synergizing with NLRP3 to accelerate podocyte aging [121].

Mitochondrial dysfunction is a key driver of podocyte aging in LN. C5a/C5aR1 promotes dynamin-related protein 1 (DRP1) phosphorylation, causing excessive mitochondrial fragmentation, ROS accumulation, oxidative DNA damage, and activation of senescence pathways [122]. Conversely, Nestin preserves mitochondrial integrity via mitophagy, reducing ROS and cellular injury [117].

Loss of cytoskeletal integrity contributes to podocyte aging in LN. Upregulated Piezo1 responds to mechanical stress by increasing Ca^2+^ influx, activating Rac1 and paxillin signaling, and driving cytoskeletal remodeling, FPE, and GFB disruption. Concurrent nephrin downregulation or mislocalization further impairs podocyte function, promotes senescence, and exacerbates proteinuria and glomerular injury [123].

In LN, podocyte aging is driven by impaired autophagy, inflammasome activation, mitochondrial dysfunction, and cytoskeletal disintegration (Figure 2 and Figure 3). Unlike DKD, where metabolic stress dominates, or MCD/MN, where adaptive immune responses are key, LN emphasizes autoimmunity-induced inflammasome activation and complement-mediated mitochondrial injury as the central drivers of senescence.

### 4.5. Hypertension/Obesity-Related Glomerulopathy

Hypertension and obesity impose unique stressors on podocytes, driving premature podocyte aging through convergent molecular pathways, manifesting as elevated p21^Cip1^, p53, and SASP expression independent of chronological age [124].

In hypertensive nephropathy (HN), podocyte aging is driven by mechanical stress, redox imbalance, aberrant protein modifications, and proteostasis disruption [125,126]. Upregulated Piezo1 transduces biomechanical strain via Ca^2+^ influx, activating Rac1 and downstream cytoskeletal remodeling and leading to FPE. While four-and-a-half LIM domains protein 2 (FHL2) amplifies Rac1 activity, destabilizing the actin cytoskeleton [127,128,129]. Concurrent angiotensin II overactivation impairs anaerobic glycolysis, causing energy deficits, mitochondrial dysfunction, and activation of senescence signaling [130]. Post-translational modifications further contribute. Ubiquitin-specific protease 22 (USP22) stabilizes high mobility group box 1 (HMGB1), enhancing angiotensin II-induced inflammation, oxidative stress, and apoptosis [131], whereas sirtuin 2 (SIRT2) preserves cytoskeletal integrity by deacetylating Septin4 [132]. Hypertensive stimuli also suppress heat shock protein 70 (Hsp70), compromising proteostasis, inducing protein aggregation and ERS, and accelerating senescence [133]. Together, these alterations impair podocyte integrity and regenerative potential, promoting chronic aging in HN.

In obesity-related glomerulopathy (ORG), podocyte aging is driven by lipid overload, chronic inflammation, and mitochondrial stress [134]. Excess lipids are internalized via CD36, intensified by phosphatase and tensin homolog (PTEN) downregulation, leading to lipotoxicity and activation of the ROS/NF-κB/NLRP3 inflammasome axis [135,136]. Chronic inflammation causes cytoskeletal disarray, mitochondrial damage, and cell cycle arrest, while reduced adiponectin further weakens antioxidant and anti-inflammatory defenses [137]. Mediators such as purinergic 2X7 (P2X7) and acid sphingomyelinase enhance inflammasome activity and propagate paracrine injury via inflammatory exosomes [138,139]. Compensatory WT1 upregulation partially maintains podocyte identity but cannot prevent progressive podocyte loss [140,141]. Dysregulated Wnt/β-catenin signaling, overactivated by aldosterone, drives dedifferentiation, mesenchymal transition, and senescence-associated gene expression [142].

Podocyte aging in HN and ORG arises from overlapping stress pathways. In contrast to FSGS and DKD, where intracellular metabolic or hyperglycemic stress predominates, HN and ORG highlight biomechanical strain and lipid-induced inflammation as the main drivers of premature podocyte aging (Figure 2 and Figure 3).

### 4.6. Genetic Podocytopathies

Genetic podocytopathies such as Alport syndrome (AS) and Fabry disease (FD) drive premature podocyte aging through hereditary defects that impair structural integrity and intracellular homeostasis [143,144] (Figure 2 and Figure 3).

In AS, podocyte aging arises from inherited GBM structural defects due to COL4A3, COL4A4, or COL4A5 mutations, which disrupt type IV collagen networks and impose chronic stress on overlying podocytes [145,146,147]. Disease progression further drives aging through oxidative stress, DNA damage, cytoskeletal disruption, and lipid stress. Key mediators include NOX4, amplifying mtROS, and matrix metalloproteinase-2 (MMP-2), degrading extracellular matrix (ECM) and weakening podocyte–GBM adhesion, leading to DDR and cell cycle arrest [148,149,150]. Loss of sphingomyelin phosphodiesterase acid-like 3b (SMPDL3b) disrupts cytoskeletal integrity and membrane dynamics, increasing susceptibility to detachment and apoptosis [151], while p53 overactivation promotes premature senescence [152]. Vascular endothelial growth factor A (VEGFA) accumulation in the GBM and discoidin domain receptor 1 (DDR1) activation link matrix abnormalities to lipid-mediated injury, further exacerbating mitochondrial damage, ERS, and autophagic impairment [153,154]. Together, inherited structural fragility and acquired stress-induced mechanisms converge to drive podocyte senescence in AS.

FD, an X-linked lysosomal storage disorder caused by α-galactosidase A deficiency, leads to globotriaosylceramide (GL-3) accumulation in podocytes, driving aging through multiple interrelated pathways [155,156,157]. Progressive GL-3 buildup correlates with podocyte loss, structural damage, and proteinuria, particularly in female heterozygous carriers [158,159]. FD podocytes exhibit ERS, chronic inflammation, and impaired autophagy, notably disrupted autophagosome–lysosome fusion, causing accumulation of damaged mitochondria and undegraded substrates, which exacerbate oxidative stress and trigger senescence [160,161,162]. Furthermore, ceria–zirconia nanoparticles have been shown to restore autophagic flux and reduce GL-3 accumulation in FD, highlighting the potential of autophagy-targeting strategies [163]. α-Synuclein accumulation contributes to cytoskeletal disorganization and FPE, while ferroptosis-related pathways enhance susceptibility to oxidative injury [164,165,166]. Integrin α3β1 dysfunction further impairs adhesion, promoting detachment and mechanical instability [167]. Collectively, GL-3 accumulation initiates a self-reinforcing cascade of autophagy impairment, cytoskeletal collapse, ferroptotic signaling, and adhesion loss, leading to sustained podocyte aging and progression of Fabry nephropathy.

Unlike acquired podocytopathies, genetic diseases feature primary hereditary defects that predispose podocytes to chronic stress, making ECM disruption and lysosomal substrate accumulation key triggers of premature aging.

## 5. Therapeutic Approaches Targeting Podocyte Aging

### 5.1. Senolytic Therapies

Senolytic therapy has emerged as a promising approach to alleviate podocyte aging. In both animal models and human studies, the senolytic combination of dasatinib and quercetin (D + Q) effectively eliminates senescent cells, reduces the SASP, and improves kidney structure and function [168] (Table 1).

Mechanistically, D + Q therapy alleviates podocyte aging by modulating key signaling pathways associated with aging and cellular stress. Quercetin, a flavonoid with antioxidant and anti-inflammatory properties, promotes autophagic clearance of damaged organelles and misfolded proteins in aged podocytes by activating AMPK signaling and inhibiting mTOR signaling [169]. In DKD models, the D + Q combination specifically activates autophagy and inhibits Notch pathway overactivation, a known inducer of podocyte dedifferentiation and senescence, thereby preserving podocyte identity and reducing proteinuria. Additionally, D + Q reduces the expression of senescence markers (p16^INK4a^ and p21^CIP1^) and improves mitochondrial integrity, thereby reinforcing cellular resilience under metabolic stress [170].

Beyond diabetic models, senolytic therapy has shown beneficial effects in acute and chronic kidney injury contexts, suggesting its broader applicability across podocytopathies [171]. In murine models of ischemia–reperfusion injury and renal fibrosis, D + Q treatment reduced senescent cells in renal tubules and glomeruli, facilitated tissue repair, and attenuated progression to CKD. These outcomes underscore the dual benefit of senolytics: both halting maladaptive aging responses and reactivating regenerative programs [172,173]. Importantly, senolytic therapy is under clinical investigation for renal aging, and combining it with autophagy enhancers or anti-fibrotics may improve outcomes in age-related podocytopathies [174]. However, a key knowledge gap remains regarding the long-term safety, optimal dosing, and tissue-specific targeting of senolytic therapy in podocytes, which must be addressed before its widespread clinical application can be made.

### 5.2. Podocyte Regeneration Strategies

Podocyte regeneration represents a novel and promising strategy to counteract podocyte aging and loss in podocytopathies. Unlike terminally differentiated podocytes, renal progenitors, including parietal epithelial cells (PECs), can differentiate into new podocytes under certain conditions [175] (Table 1).

#### 5.2.1. Mechanisms Underlying Podocyte Regeneration

In models of FSGS, DKD, and AS, regenerative mechanisms were observed to restore podocyte number and function, contributing to glomerular remission [176,177]. Moreover, genetic reprogramming strategies using stem cells or enhancing podocyte-specific metabolic regulators such as PKM2 can promote podocyte regeneration and restore glomerular integrity [178]. Mechanistically, podocyte regeneration is orchestrated by a complex interplay between local cues, epigenetic regulation, and chemokine-mediated feedback circuits. For example, C–X–C Motif Chemokine Ligand 12 (CXCL12) blockade was shown to disrupt inhibitory signals between podocytes and their progenitors, enabling PECs to repopulate lost podocytes specifically in cortical nephrons [179]. Additionally, signals from injured podocytes may act as feedback to recruit and induce progenitor cell differentiation. Other pathways, such as Notch inhibition and WT1 activation, have also been implicated in directing progenitor cell fate toward a podocyte phenotype [180]. These mechanistic insights provide a rationale for pharmacological and genetic strategies aimed at enhancing podocyte regeneration.

#### 5.2.2. Drug-Based Interventions for Podocyte Regeneration

Retinoic acid derivatives and angiotensin receptor blockers (ARBs) promote podocyte regeneration, counteracting aging and injury in podocytopathies. All-trans retinoic acid (ATRA) enhances podocyte differentiation by activating retinoic acid receptor α (RARα), upregulating nephrin and podocin to improve glomerular structure and function [181]. In models of nephrotoxic serum-induced glomerulonephritis, retinoic acid treatment attenuates glomerular injury and fosters podocyte regeneration, although proteinuria can limit this effect by sequestering retinoic acid and impairing its availability [182,183]. Concurrently, RAAS inhibition via ARBs has been found to enhance the differentiation of renal progenitor cells and renin–lineage cells into functional podocytes [184]. These interventions restore podocyte numbers, improve GFB integrity, and mitigate the progression of podocytopathies.

#### 5.2.3. Genetic and Tissue Engineering Approaches

Gene-editing technologies, such as CRISPR/Cas9 lineage tracing, have further clarified the origin and potential of podocyte progenitors, providing insights into pharmacological reprogramming [185]. Tissue engineering approaches using organoids and kidney-on-chip models are being developed to test regenerative therapies in a controlled and patient-specific context [186,187].

Despite the promise of podocyte regeneration as a therapeutic strategy, several challenges remain before podocyte regeneration can be translated into a viable therapy. First, the regenerative capacity of endogenous progenitors declines with age and chronic injury, limiting the therapeutic window. Second, inappropriate or incomplete differentiation can result in maladaptive phenotypes, including hyperplastic PECs that contribute to sclerosis. Lastly, standardized pharmacological agents or gene therapies capable of safely and efficiently activating regenerative programs are still under development. Despite these hurdles, the approaches to pharmacologically enhance or engineer podocyte regeneration exhibit significant potential in treating age-related and progressive podocytopathies [180].

### 5.3. Drug Repurposing

Drug repurposing offers a pragmatic and efficient avenue to identify therapies that mitigate podocyte aging, particularly by leveraging the known safety profiles of existing compounds. Several widely used medications have demonstrated anti-aging and renoprotective effects in preclinical models of podocytopathies (Table 1).

#### 5.3.1. Metformin and Rapamycin

Metformin and rapamycin exert protective effects against podocyte aging primarily through modulation of the AMPK/mTOR signaling axis, enhancement of autophagy, and improvement in mitochondrial function. Metformin activates AMPK, which, in turn, inhibits mTORC1, restoring autophagic flux by promoting the ULK1-initiated autophagy complex. This reduces the accumulation of damaged organelles and mitigates oxidative stress-induced senescence [188]. Additionally, metformin enhances mitochondrial biogenesis and function via the AMPK-PGC1α pathway, lowering ROS production and improving cellular energy metabolism [189]. Rapamycin, as a direct mTORC1 inhibitor, synergistically enhances autophagy, alleviates protein synthesis burden, and reduces podocyte aging [190]. In DKD and idiopathic MN, the combination of metformin and rapamycin has been shown to restore podocyte morphology and GFB integrity, significantly reducing proteinuria. Together, these agents target key stress and aging pathways in podocytes, offering a promising strategy to delay or reverse senescence-associated podocyte injury.

#### 5.3.2. Losartan

Losartan, an ARB widely used in the treatment of hypertension and DKD, exerts renoprotective effects beyond hemodynamic regulation by upregulating Hsp70 in podocytes. Hsp70 acts as a molecular chaperone, maintaining proteostasis, enhancing stress resistance, and stabilizing nephrin and podocin. Through this mechanism, losartan mitigates oxidative stress and ERS, reduces NF-κB-mediated inflammatory signaling, and preserves mitochondrial function in podocytes. These protective effects preserve podocyte structure and function, reduce FPE and detachment, mitigate podocyte aging, and ultimately slow the progression of podocytopathies [133].

#### 5.3.3. Canagliflozin

Likewise, canagliflozin was shown to exert protective effects on podocytes through immunomodulatory and autophagy-enhancing mechanisms. Specifically, canagliflozin corrected the Th1/Th2 imbalance by promoting Th1 responses and suppressing Th2-mediated inflammation, thereby reducing the autoimmune burden associated with MN. Simultaneously, it restored autophagic activity in podocytes by activating the ULK1 complex and suppressing mTOR signaling, leading to increased expression of autophagy markers such as LC3-II and Beclin1. These effects resulted in improved podocyte structural integrity, reduced cellular senescence, decreased proteinuria, and attenuation of glomerular injury, indicating that canagliflozin mitigates podocyte stress and senescence-related inflammation in MN [191].

#### 5.3.4. Curcumin

Curcumin, a natural polyphenolic compound with antioxidant and anti-inflammatory properties, has shown promising protective effects against podocyte aging in both DKD and ORG. In DKD, curcumin directly targets the chemokine CXCL16, reducing lipid accumulation, oxidative stress, and pro-inflammatory cytokine production in podocytes. This inhibits NLRP3 inflammasome activation and alleviates lipotoxic damage, restoring autophagic and metabolic homeostasis [109]. In ORG, curcumin suppresses aberrant activation of the Wnt/β-catenin signaling pathway, thereby preserving the expression of nephrin and podocin, preventing FPE and proteinuria [192]. Through these mechanisms, curcumin attenuates podocyte aging and helps maintain GFB integrity across multiple podocytopathies.

These findings underscore that drug repurposing not only accelerates the translation of anti-aging strategies into the clinic but also broadens therapeutic options for combating podocyte aging in diverse podocytopathies. Nonetheless, critical questions remain about how these repurposed agents influence podocyte-specific aging pathways in the long term. Their combinatorial effects and safety profiles also warrant further preclinical and clinical evaluation to maximize efficacy.

**Table 1:** Potential therapeutic approaches targeting podocyte aging.

Therapeutic Strategies	Example	Mechanisms	Diseases	Reference
Senolytic therapies	Dasatinib and quercetin (D + Q)	Enhancing autophagic activity through activating AMPK and suppressing mTOR signaling; activating autophagy, inhibiting Notch pathway overactivation, and improving mitochondrial integrity.	DKD	[169,170]
Podocyte regeneration	Retinoic acid	Enhancing podocyte differentiation by activating retinoic acid receptorα (RARα).	/	[181]
	Angiotensin receptor blockers (ARBs)	Enhancing the differentiation of renal progenitor cells and renin–lineage cells into functional podocytes.	FSGS	[184]
	CRISPR/Cas9-based lineage tracing	Cellular reprogramming.	/	[185]
	Tissue engineering	Organoids and kidney-on-chip models.	/	[186,187]
Drug repurposing	Metformin	Activating AMPK, restoring autophagic flux by promoting the ULK1-initiated autophagy complex, and enhancing mitochondrial biogenesis and function via the AMPK-PGC1α pathway.	DKD, MN	[188,189]
	Rapamycin	Inhibiting mTORC1, enhancing autophagy, and alleviating protein synthesis burden.	DKD, MN	[190]
	Losartan	Upregulating Hsp70; mitigating oxidative stress and ERS, reducing NF-κB-mediated inflammatory signaling, and preserving mitochondrial function.	DKD	[133]
	Canagliflozin	Correcting the Th1/Th2 imbalance; restoring autophagic activity by activating the ULK1 complex and suppressing mTOR signaling.	MN	[191]
	Curcumin	Targeting CXCL16, reducing lipid accumulation, oxidative stress, and pro-inflammatory cytokine production; suppressing aberrant activation of the Wnt/β-catenin signaling pathway.	DKD, ORG	[109,192]

## 6. Conclusions and Perspectives

Podocyte aging has emerged as a critical contributor to the onset and progression of diverse podocytopathies. Although substantial advances have been made in elucidating the molecular underpinnings, ranging from cellular senescence and mitochondrial dysfunction to epigenetic alterations, the field still faces considerable challenges. One major hurdle lies in distinguishing the causative mechanisms of aging from secondary changes associated with disease progression. Additionally, the lack of reliable in vivo markers and standardized models of podocyte aging hampers mechanistic studies and therapeutic validation.

Future research should prioritize several compelling directions. Single-cell and spatial transcriptomics may offer unprecedented resolution in profiling aging podocytes within the complex kidney microenvironment. Integrating these omics with epigenetic and metabolic profiling could unravel novel targets and aging-specific vulnerabilities in podocytes. Moreover, emerging strategies such as precision senolytic therapies, reprogramming techniques to restore podocyte plasticity, and organoid-based drug screening platforms are poised to reshape therapeutic landscapes. Importantly, future studies should also address how systemic factors (e.g., immune aging, metabolic imbalance, and gut–kidney axis alterations) interface with podocyte aging.

In conclusion, advancing our understanding of podocyte aging holds significant potential for developing age-adapted, mechanism-based interventions in podocytopathies. Overcoming current limitations will require a multidisciplinary effort, combining basic science innovations with translational and clinical research to achieve meaningful progress in podocytopathy management.

## Data Availability

Not applicable.
