# Correction: Ma et al. Mechanisms and Therapeutic Perspectives of Podocyte Aging in Podocytopathies. *Int. J. Mol. Sci.* 2025, *26*, 9159

**DOI:** 10.3390/ijms27198660

**Published:** 2026-09-28

**Authors:** Si-Jia Ma, Yu-Ting Zhu, Fang-Fang He, Chun Zhang

**Affiliations:** Department of Nephrology, Union Hospital, Tongji Medical College, Huazhong University of Science and Technology, Wuhan 430022, China; masijia1018@163.com (S.-J.M.); drzhuyuting@hust.edu.cn (Y.-T.Z.)

The following reference [189] was retracted and must be removed from the original publication [1]:

189. Sun, T.; Liu, J.; Xie, C.; Yang, J.; Zhao, L.; Yang, J. Metformin Attenuates Diabetic Renal Injury via the AMPK-Autophagy Axis. *Exp. Ther. Med.* **2021**, *21*, 578. https://doi.org/10.3892/etm.2021.10010.

The scientific content of [189] could not be replaced with any other publication. Therefore, the authors decided to remove reference [189]. With this correction, the order of some references has been adjusted accordingly.

The authors state that the scientific conclusions are unaffected. This correction was approved by the Academic Editor. The original publication has also been updated.
